# Translation and cultural adaptation of the health literacy questionnaire (HLQ) in Morocco

**DOI:** 10.1186/s41687-026-01111-3

**Published:** 2026-06-11

**Authors:** Karima Boumendil, Younes Iderdar, Nadia Al Wachami, Maryem Arraji, Salwa Najmi, Melanie Hawkins, Richard Osborne, Mohamed Chahboune

**Affiliations:** 1 Present Address: Hassan First University of Settat, Higher Institute of Health Sciences, Laboratory of Health Sciences and Technologies, Settat, Morocco; 2 Higher Institute of Nursing Professions and Health Techniques, Tiznit, Morocco; 3 Icahn School of Medicine at Mount Sinai, New York, USA; 4 Global Health and Equity Development Hub. La Trobe Rural Health School, La Trobe University, Victoria, Australia

**Keywords:** Health literacy, Translation, Morocco, Health literacy questionnaire (HLQ), Non-communicable diseases, Validation

## Abstract

**Background:**

Health literacy is an established determinant of health and a key tool in the development of public health interventions. It plays an important role in improving the health and equity of individuals and populations. The Health Literacy Questionnaire (HLQ) is a widely used multidimensional instrument designed to assess various domains of health literacy across diverse populations. However, no culturally and linguistically adapted version of the HLQ is currently available for the Moroccan context, where Moroccan Arabic (Darija) is the primary language of everyday communication, including interactions with healthcare professionals. Therefore, the objective of this study was to carry out the socio-cultural and linguistic adaptation of the HLQ into Darija.

**Methods:**

The HLQ was culturally and linguistically adapted for Morocco through a collaborative process with the HLQ developers, community members, healthcare professionals, and the project team from the Higher Institute of Health Sciences (ISSS). Forward and backward translation and cognitive interviews with 28 primary healthcare users recruited from two Moroccan provinces (Kenitra and Settat) maximized that each item reflected its intended meaning. Data were analyzed using thematic analysis to refine the adapted version for the Moroccan context.

**Results:**

The 28 participants (71.4% women) were aged 18 to 70, with diverse educational backgrounds and resided in both urban and rural areas. Of the 44 translated items, 19 (43%) required further linguistic or cultural adaptation for Darija speakers (Moroccan Arabic dialect). Participants generally understood and responded to the items per each item’s intent, but qualitative analysis highlighted important nuances. A marked dependence on medical authority appeared to limit participants’ ability to manage their own health and was associated with persistent difficulties navigating the healthcare system and critically appraising health information. In contrast, items related to social support were more readily understood, highlighting the central role of family networks. Collectively, these findings underscore the interplay between cultural norms and structural barriers in shaping perceptions of health literacy in Morocco.

**Conclusion:**

The cognitive and cultural adaptation of the HLQ into Moroccan Darija supported the relevance of the nine scales and provided insight into locally grounded conceptions of health. This process highlights the importance of a participatory and context-sensitive approach to achieving conceptual fidelity and cultural appropriateness. However, it should be emphasized that this study represents an initial yet essential step in the overall validation process. While the adapted Darija version of the HLQ cannot yet be considered a fully validated instrument for use in the general population, it provides a robust foundation for subsequent psychometric evaluation. Further research is required to evaluate its psychometric properties, including internal structure and reliability, to determine its suitability for assessing health literacy in the Moroccan context.

**Plain English Summary:**

This study is important because health literacy development is critical to helping individuals and populations access and use health information and services to manage their health. The first step in measuring health literacy is to adapt tools to local languages and cultures. The study focuses on adapting the Health Literacy Questionnaire (HLQ) to the Darija language (Moroccan Arabic dialect) so that the health literacy needs of people in primary healthcare settings in Morocco can be assessed. The methods used in the study helped ensure that the Darija HLQ is relevant and understandable in the Moroccan context. The study found that the Darija HLQ was well understood by most users, though some with lower education levels found certain questions unclear. This means that the tool is adequate for use in Morocco, but further refinement may be needed for certain groups. The questionnaire was translated and adapted through a collaborative translation process to maximize its cultural and linguistic appropriateness for Moroccan users.

**Supplementary Information:**

The online version contains supplementary material available at 10.1186/s41687-026-01111-3.

## Background

Health literacy predicts health outcomes. It has become a crucial concept for guiding interventions to improve health and health equity among individuals and communities, especially during interactions between healthcare providers and community members (1).

According to the World Health Organization (WHO), health literacy refers to “*The cognitive and social skills that determine the motivation and ability of individuals to gain access to, understand, and use information in ways which promote and maintain good health”* [2]. This knowledge and these skills are enhanced by organizational structures and resource availability, enabling individuals to access, understand, appraise, remember and use information in ways that promote and maintain their health and well-being, as well as the health of those around them [3]. This definition highlights the multidimensional nature of health literacy, expanding its scope beyond merely reading and comprehending health information, which was the focus of early measurement tools [4]. It emphasizes the importance of health promotion and the social resources and skills needed to navigate complex healthcare systems [3].

The development of health literacy is essential to addressing the global burden of non-communicable diseases (NCDs), including cancer, diabetes, chronic respiratory diseases, and cardiovascular conditions [5]. Although health literacy is increasingly recognized internationally, only a limited number of countries have fully integrated it as a key programmatic component of their national health strategies [6, 7]. In some cases, it is only briefly mentioned under broader public health objectives [8]. This gap is particularly evident in Morocco, where published studies on health literacy are rare.

Measuring multiple, actionable dimensions of health literacy is a crucial step in informing actions and strategies to address health inequities [9]. However, existing tools for measuring health literacy have primarily been based on Western conceptions of health and literacy, which may not align with the perspectives of all study participants [3]. Additionally, the context and values of the tool’s developers, as well as the underlying definitions of the concept being measured and the model used to construct it, inevitably influence the development of such measurement tools [10, 11].

The Health Literacy Questionnaire (HLQ) [9] is one of the most widely used health literacy tools in public health, health promotion, and healthcare services research. The HLQ is being used in over 80 countries, with translation into 30 language versions and further translations in progress [11]. In Europe, existing translations include Danish [10], Slovakian [12], German [13], Swedish [14], Norwegian [15], French [16], and Portuguese [17]. In Asia, there are translations in Arabic [18], Nepalese [19], Korean [20], Vietnamese [21], and in African countries, there are translations into Bambara (Mali) [22], Yoruba (Ghana) [23], and Arabic (Egypt) [24].

Validity testing of the HLQ remains limited in the Arab world and is almost nonexistent in North Africa, particularly in Morocco. Moreover, the available versions are mainly based on Standard Arabic and have been tested in Middle Eastern contexts, without considering local linguistic and cultural specificities, including dialects such as Darija.

Indeed, in Morocco, the epidemiological shift towards non-communicable diseases (NCDs), which account for more than 80% of deaths, is accompanied by increasingly complex healthcare needs requiring high levels of health literacy [25, 26]. However, this requirement is hampered by persistent structural constraints, including an under-resourced healthcare system, marked territorial inequalities, and significant socioeconomic disparities [27–29]. Adding to these challenges is the linguistic gap between the population and the healthcare system. While Darija (Moroccan Arabic) is the predominant language spoken by 92% of the population, compared to 25% for Amazigh varieties [30], medical interactions, professional training, and informational materials remain primarily in French or Standard Arabic [16, 31, 32].

This linguistic asymmetry establishes a communication hierarchy that limits understanding of health information, hinders patient engagement in care, and undermines their ability to act on their health. [33, 34]. These difficulties are particularly exacerbated in rural areas and among Amazigh-speaking populations, especially women, where linguistic and educational barriers combine to exacerbate inequalities in access to and use of health services [35, 36]. Although traditionally oral, Darija is gaining ground in written form in informal contexts (social media, advertising, youth communication), enabling culturally relevant and accessible messages [37]. In this context, the absence of a validated, multidimensional health literacy measurement tool adapted linguistically and culturally to Darija, such as the HLQ, constitutes a major methodological gap [38]. It limits the ability to reliably assess health literacy levels among the Moroccan population, identify vulnerable groups, and guide interventions tailored to the social determinants of health. This study aimed to translate the HLQ into Darija to maximize cultural relevance, particularly for people affected by NCDs, and to help maximize its potential to inform the development of interventions for chronic disease control, prevention, and health promotion.

## Materials and methods

A cross-sectional descriptive study was conducted using face-to-face administration of the Darija version of the Health Literacy Questionnaire (HLQ). Cognitive interviews were conducted to examine how respondents understood HLQ concepts and interpreted the intended meaning of each item. The interviews were conducted between January and March 2024 at four primary healthcare facilities (two in rural areas and two in urban areas) across two provinces of Morocco with predominantly Darija-speaking populations (Settat in the Casablanca-Settat region and Kenitra in the Rabat-Sale-Kenitra region). These regions, among the most densely populated in Morocco, are characterized by significant disparities in resources and living standards.

The inclusion criteria were individuals aged 18 and older who were fluent in Darija and seeking vaccinations, being monitored for a chronic illness (diabetes, hypertension, etc.), or accessing other primary healthcare services.

A diverse sample was necessary to assess whether individuals from various cultural and linguistic backgrounds interpreted the HLQ items as intended. People who did not provide consent or whose health did not permit it were excluded.

### The health literacy Questionnaire (HLQ)

The HLQ consists of 44 items organized into nine health literacy scales. Each scale contains 4 to 6 items (Table 1).

**Table 1:** The health literacy Questionnaire (HLQ) scales with descriptions of higher and lower scores (Osborne et al. 2021)

Lower HLQ scale scores		Higher HLQ scale scores
**Part 1: Scales 1 to 5** **Score range: 1 to 4 (1 = Strongly disagree, 2 = Disagree, 3 = Agree, 4 = Strongly agree)**		
**1. Feeling understood and supported by healthcare providers** (4 items)		
Is unable to engage with doctors and other healthcare providers. The person doesn’t have a regular healthcare provider and/or has difficulty trusting healthcare providers as a source of information and/or advice.		Has an established relationship with at least one healthcare provider who knows them well. The person trusts this provider to give useful advice and information and to assist them to understand information and make decisions about their health.
**2. Having sufficient information to manage my health** (4 items)		
Feels that there are many gaps in their knowledge and that they don’t have the information they need to live with and manage their health concerns.		Feels confident that they have all the information that they need to live with and manage their condition and to make decisions.
**3. Actively managing my health** (5 items)		
Doesn’t see their health as their responsibility. The person is not engaged in their health care and regards health care as something that is done to them.		Recognises the importance of and can take responsibility for their health. The person proactively engages in their care and makes their own decisions about their health.
**4. Social support for health** (5 items)		
Completely alone and unsupported.		The person’s social system provides them with all the support they want or need.
**5. Appraisal of health information** (5 items)		
No matter how hard they try, the person cannot understand most health information and becomes confused when there is conflicting information.		Is able to identify good information and reliable sources of information. They can resolve conflicting information by themselves or with help from others.
**Part 2: Scales 6 to 9** **Score range: 1 to 5 (1 = Cannot do or always difficult, 2 = Usually difficult, 3 = Sometimes difficult, 4 = Usually easy, 5 = Always easy)**		
**6. Ability to actively engage with healthcare providers** (5 items)		
Is passive in their approach to health care, inactive, does not seek or clarify information and advice and/or service options. Accepts information without question. Unable to ask questions to get information or to clarify what they don’t understand. Accepts what is offered without seeking to ensure it meets their needs. Feels unable to share concerns.		Is proactive about their health and feels in control in relationships with healthcare providers. Is able to seek advice from additional healthcare providers when necessary. Keeps going until they get what they want. Empowered.
**7. Navigating the healthcare system** (6 items)		
Is unable to advocate on their own behalf and unable to find someone who can help them use the healthcare system to address their health needs. Does not look beyond obvious resources and has a limited understanding of what is available and what they are entitled to.		Is able to find out about services and supports so they get all their needs met. Able to advocate on their own behalf at the system and service level.
**8. Ability to find good health information** (5 items)		
Cannot access health information when required. Is dependent on others to offer information.		Is an ‘information explorer’. Actively uses a diverse range of sources to find information and is up to date.
**9. Understanding health information well enough to know what to do** (5 items)		
Has problems understanding any written or spoken health information or instructions about treatments or medications. Unable to read or write well enough to complete medical forms.		Can understand all written and spoken information (including numerical information).

### Translation process

The translation and cultural adaptation were carried out in accordance with the Translation Integrity Protocol (TIP), a methodology recommended by the designers of the HLQ and used in existing translations of this instrument [13, 39–41]. The translation team sought to ensure that the translated questions were worded in accordance with the descriptions of item intents of the original English HLQ. Cognitive interviews provide data on response processes, which is an important form of validity evidence [42]. These interviews assess whether participants engage with, reflect on, and respond to the translated content as intended. After receiving the license (TL2103IS 17/09/2021) to adapt the HLQ, we implemented the TIP.

The process included forward translation, backward translation, and translation-consensus discussions, which served as a group cognitive process. The translation was documented in the TIP translation management grid (Supplementary material 1).

**Fig. 1:**
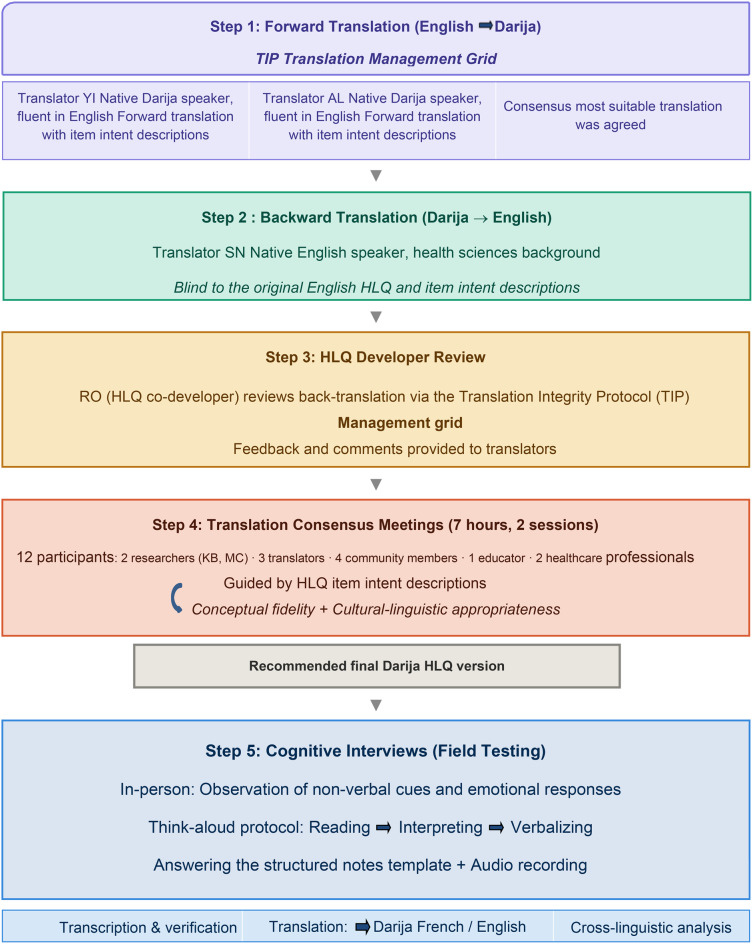
HLQ translation and cultural adaptation process into Moroccan Darija, informed by the TIP management grid framework (Osborne & Hawkins)

1. Two translators, YI and NE, both native Darija speakers fluent in English, are healthcare professionals with advanced academic degrees (a Master’s in advanced care and a PhD in Epidemiology) and extensive research experience. They performed the forward translation from English to Darija, guided by the HLQ item intent descriptions. After completing the TIP process, the translators submitted their versions, and they reached an agreement on the best translation through discussion.
2. A native English speaker (SN), with strong proficiency in the target language and experience in biomedical contexts, and fluent in Darija, conducted the blind backward translation.
3. Using the translation management grid, which included comments from the forward and backward translators, one of the HLQ developers (RO) reviewed the back translation and provided comments for the translators.
4. A translation consensus meeting was held to confirm the HLQ for testing in cognitive interviews (Figure 1).

Two consensus meetings, totaling seven hours, were held after the forward and backward translations and HLQ developer feedback. Twelve participants, including two researchers (KB, MC), three translators, four community members, one educator, and two healthcare professionals, discussed the intent and cultural relevance of the items. The representative from the tool development team (RO) made a significant contribution by clarifying the intent of the items and resolving disagreements. It was confirmed that the Darija version items were semantically equivalent to the original English instrument and potentially appropriate for people with the most diverse language skills and experiences in accessing, understanding, and using health information and services, thereby preserving conceptual fidelity and cultural-linguistic appropriateness.

A recommended pre-final version of the translation was tested in the field. Cognitive interviews were conducted in person, allowing interviewers to observe participants’ nonverbal cues and emotional responses as they read, interpreted, verbalized their thoughts about, and answered the translated items. Interviewers used a structured template to take notes during the sessions, which were audio-recorded. After transcription, the recordings were reviewed for accuracy, and necessary corrections were made. Transcripts were then translated from Darija into French and English for cross-linguistic analysis.

The data were organized into the HLQ’s nine domains and analyzed using an inductive thematic approach [41] grounded in the HLQ constructs. This dual method enabled themes to be identified directly from participants’ Darija responses while ensuring that interpretations remained aligned with each item’s intended meaning. The analysis assessed whether the Darija versions conveyed the same concepts as the original English HLQ and whether participants’ interpretations aligned with the HLQ scale constructs. Textual quotations in Darija were retained to provide direct evidence of understanding and conceptual alignment, highlighting linguistic nuances and contextual factors, including socioeconomic constraints. Coding was performed manually using detailed interview notes and summaries, with a color-coding system to organize emerging themes and facilitate comparisons across transcripts. Particular care was taken to identify linguistic challenges, cultural ambiguities, and conceptual misunderstandings to inform potential improvements to item wording [40]. The qualitative data were coded by the interviewer in collaboration with researchers from the Hassan I University of Settat team. This phase of translation and cultural adaptation is a preliminary yet crucial step, laying the groundwork for the subsequent quantitative psychometric validity testing of the Darija version of the HLQ.

### Ethics approval

Ethical approval for this study was granted by the biomedical research ethics committee at the Faculty of Medicine and Pharmacy of Rabat, Mohamed V University, Morocco (Approval Number: CERB 68–23). All procedures were conducted in accordance with the General Data Protection Regulation and the ethical guidelines established by the Declaration of Helsinki.

All participants provided informed consent to participate in the survey and interviews. The information letter also clarified that participation was voluntary and that participants could withdraw at any time. To ensure confidentiality and anonymity, each participant was assigned a pseudonym.

### Data collection

Cognitive interviews were conducted in accordance with the protocol established by Hawkins and Osborne [43] for testing HLQ items with a target population. The protocol recommends testing each item with four to six participants and analyzing their responses to verify understanding of the concept being measured. Each item was reviewed by at least six participants, which corresponds to the upper limit of the recommended range.

After giving their consent, literate individuals received a printed questionnaire, while those unable to read independently (three with no formal schooling and three with insufficient primary literacy, *n* = 6) had the questions read aloud by the interviewer in Darija, consistent with standard HLQ administration procedures for low-literacy populations. This approach ensured full participation by all individuals, regardless of literacy level, reflecting the instrument’s intended applicability to diverse populations.

The interviewer (KB) explained that there were no right or wrong answers and emphasized that the objective was to explore participants’ understanding of the questions and their experiences with health services and daily life. Wherever possible, information was presented objectively. Participants were asked to read the questions aloud and explain their understanding, allowing the interviewer to identify any uncertainty or difficulty. This approach aimed to identify necessary modifications to the Darija text. In addition to the HLQ, participants were asked to provide sociodemographic information (age, sex, marital status, education level, living conditions, and employment status), as well as a question about their chronic disease status and the type of social security coverage they receive.

## Results

### Settings and population

The interviewees, aged 18 to 70 years, had varying levels of education and socioeconomic status. Because the full questionnaire contained 44 questions, not all participants answered every question due to fatigue. In total, 15 participants answered all questions, while the remaining 13 answered only some.

Table 2 presents the demographic characteristics of the interviewees. Of the 28 patients (median age 39; SD = 14), 22 were women, 16 had not completed secondary education, 11 lacked medical insurance, and 8 reported chronic illnesses, including asthma, diabetes, hypertension, renal failure, or hyperthyroidism.

**Table 2:** Sociodemographic characteristics of the interviewed participants (*N* = 28)

Variable	Category	n (%)
**Sex**	Male	6 (21.4)
	Female	22 (78.6)
**Age (years)**	≤40	16 (57.1)
	>40	12 (42.9)
**Educational level**	No formal education	3 (10.7)
	Primary	7 (25.0)
	Secondary	6 (21.4)
	University	12 (42.9)
**Marital status**	Single	7 (25.0)
	Married	19 (67.9)
	Divorced	2 (7.1)
**Area of residence**	Urban	20 (71.4)
	Rural	8 (28.6)
**Occupational status**	Employed (full/part-time)	18 (64.3)
	Not employed*	10 (35.7)
**Chronic disease**	Yes	7 (25.0)
	No	21 (75.0)
**Medical insurance**	Public	11 (39.3)
	Private	6 (21.4)
	None	11 (39.3)

*Not employed includes housewives, unemployed, and retired participants

Cognitive interviews revealed that item comprehension varied systematically by education level, residence (rural or urban), occupation, and healthcare experience. Participants with lower educational attainment had particular difficulty with an item in Scale 5. Appraisal of health information, “5.12.I always compare health information from different sources and decide what is best for me” and items of the Scale 8. Ability to find good health information. Rural residents often misinterpreted an item from Scale 7. Navigating the healthcare system, “7.1. Find the right health care” as referring to relational intermediaries rather than formal procedures. Full-time homemakers and unemployed individuals reinterpreted an item from Scale 3. Actively managing my health, “3.6.I spend quite a lot of time actively managing my health” as reactive illness management. Participants with chronic illnesses relied heavily on healthcare professionals to understand health information, despite greater practical familiarity with it. These findings support the cultural validity of the adapted HLQ by showing that comprehension challenges were distributed across a diverse sample rather than confined to a specific subgroup.

### Translation and adaptation consensus in Darija

The majority of items did not induce comprehension difficulties or hesitation during the interviews. Participants frequently responded immediately and, subsequently, provided spontaneous elaborations in their own words, in ways that aligned closely with the intended meaning of the items. However, to ensure linguistic and cultural equivalence, 19 of the 44 HLQ items in Darija needed modifications (Table 3). These modifications encompassed lexical translation, semantic reformulation, syntactic simplification, and cultural adaptation. The extent of adjustments varied among the different scales. The scales requiring the most revisions were those pertaining to engagement with health professionals: “6. Ability to actively engage with healthcare providers” (*n* = 12), “7. Navigation in the health system” (*n* = 13), and “9. Understanding health information well enough to know what to do” (*n* = 11).

**Table 3:** HLQ items requiring linguistic and cultural adjustments, identified through cognitive interviews (*n* = 28)

Scale	Scale Title	Adjusted item or Concept	Item(s) Concerned	Participant Feedback
S1	Feeling understood and supported by healthcare providers (HPS)	‘Healthcare provider’ misunderstood/‘rely on’ culturally incongruent	Part 1- Q2	“لا ما عندي تا واحد” (I have no one); “how come? only on doctors.”
			Part 1- Q22	
		Relational formulation: discussion with the provider perceived as inaccessible or inappropriate	Part 1- Q8	“كاين غير الخوض صحاب الصحة” (Anger & frustration at the healthcare system)
S2	Having sufficient information to manage my health (HSI)	Absolute/self-sufficient knowledge framing incompatible with a fatalistic worldview (‘Only God knows/decides’)	Part 1- Q1	“Health information is hard to find.”
			Part 1- Q14	“Not always.”
			Part 1- Q23	“Not everyone”
S3	Actively managing my health (AMH)	‘Dedicate time to health’: proactive model conflicts with reactive illness-driven behavior	Part 1- Q6	“Nothing but when bad things happened, we do.”
		‘Goals’ and ‘Fitness’: abstract planning concepts poorly understood; fitness conflated with dietary adjustment	Part 1- Q9	“I made an adjustment, like reducing an ingredient in tea,”; “eating what doesn’t make me sick.”
			Part 1- Q18	
S4	Social support for health (SS)	Confusion between moral/emotional support and financial support; reference to God as the primary source of support	Part 1- Q11	“واش فلوس؟” (Is it money?); “You should depend on yourself.”
			Part 1- Q19	
S5	Appraisal of health information (CA)	‘Sources’: concept of consulting multiple sources seen as confusing or delegated to the doctor	Part 2- Q4	“You will be disturbed in your mind … You should consult your doctor.”;
			Part 2- Q12	“Information regarding illness or health?”
		“Health information’ reframed as ‘information about illness/disease’ (reactive, not preventive frame)	Part 2- Q7	“I choose what is right for me after getting sick.”
			Part 2- Q12	“I’m illiterate.”
S6	Ability to actively engage with healthcare providers (AE)	‘Discuss’ replaced by ‘Ask’: sustained patient-provider dialogue incongruent with hierarchical consultation norms	Part 2- Q7	“What should we discuss with them? Ask them a little.”
			Part 2- Q15	“No, I don’t.”
		Cultural restraint in expressing pain or health concerns to providers	Part 2- Q4	“You can’t complain about them; you keep that between you and yourself” “It will never be easy.”
			Part 2- Q2	
S7	Navigating the healthcare system (NHS)	Concept of ‘health services’ and patients’ entitlements are unknown or inaccessible in practice	Part 2- Q16	“I didn’t understand.”
			Part 2- Q19	“I need to ask.”
		System access dependent on social relations (‘الوراق’/intermediary); navigation perceived as relational, not procedural	Part 2- Q1	“L’accueil الوراق” (the intermediary); “if there are people to help, we want it badly”; “it’s a problem.”
			Part 2- Q11	
S8	Ability to find good health information (FHI)	Language barrier: health information is often available only in French or Modern Standard Arabic, not Darija	Part 2- Q14	“Doctors talk in Arabic”; “I understand if they are in Arabic”; “We need people to explain to us.”
			Part 2- Q12	
		‘Health information’ reformulated: participants narrowly interpret as medical consultation, not self-directed information seeking	Part 2- Q18	“It’s a necessity to consult doctors”; “limited only to doctors’ information”; “I don’t look about it.”
			Part 2- Q10	
S9	Understand health information well enough to know what to do (UHI)	Administrative/medical forms filled by the doctor, not the patient (role delegation institutionally embedded)	Part 2- Q5	“No, we can’t fill them; the doctor will fill them.”
		Role of pharmacist/provider in mediating comprehension of medication labels and instructions	Part 2- Q17	“expressions they used are hard”; “only god knows what is better for me.”
			Part 2- Q9	

*Note. Items are grouped by scale. Participant feedback is reproduced verbatim from cognitive interview probing. Darija quotations are transliterated where necessary. S = Scale; HPS = Feeling understood and supported by healthcare providers; HSI = Having Sufficient Information; AMH = Actively Managing Health; SS = Social Support; CA = Critical Appraisal; AE = Active Engagement; NHS = Navigating the Healthcare System; FHI = Finding Health Information; UHI = Understanding Health Information*

The most discussed terms included “Healthcare provider”, “discuss”, “rely on”, “health information”, “sources”, and “manage”. Their translation required careful contextualization, considering social realities, healthcare interaction norms, and varying literacy levels across the population. For example, the term “discuss” was replaced with “ask” (”نسول”) to better reflect the nature of exchanges in medical consultations, which are often marked by relational asymmetry.

### Perception and adaptation of HLQ scales in the Moroccan context

Qualitative findings from cognitive interviews provided insights into the relevance and clarity of the nine HLQ scales among the Moroccan population. In general, participants demonstrated good comprehension, though several linguistic and cultural nuances emerged.

#### Feeling understood and supported by healthcare providers

Participants generally demonstrated an understanding of the items in this scale and expressed a high level of confidence in healthcare providers, indicative of profound respect for medical authority. The recurrent use of the phrase “نعوَّل/rely on” signified a strong trust in providers while also implying a form of passive reliance within the care relationship. Interactions were frequently characterized as unidirectional, with minimal dialogue. The term “health professional” was viewed as abstract and was consequently translated into the more familiar Darija expression “الناس د الصحة” (“people of health”), which was better understood and perceived as more inclusive. Nevertheless, the intended notion of freely articulating support or engaging in dialogue with health professionals did not emerge spontaneously.

#### Having sufficient information to manage my health

Participants interpreted the items in this scale in concrete terms, primarily referring to prescriptions or direct medical advice. The concept of “health information” required simplification, from “Information about health” to “معلومات على الصحة,” to improve accessibility. Although many participants reported using online sources, most relied on physicians and pharmacists, indicating that perceived availability of information does not necessarily mean they feel they have sufficient information to manage their health.

#### Actively managing my health

The items in this scale were perceived as moderately challenging overall, though many items showed strong comprehension. Responses to this item indicated that participants understood the concept of personal responsibility for health, and Darija’s formulations effectively conveyed nuances of self-management and agency. Expressions such as “صْحْتي” (ṣeḥti, my health), “تهلا فصحتك” (take care of your health), and “ندّيها فصحْتي” (look after my health) were readily understood and spontaneously rephrased by respondents, confirming the intended focus on personal engagement in health care. Participants paraphrased the item on dedicating time to health care (Scale 3, item 6) as “كناخد وقتي باش نديها فصحتي” or “كنخصص بزاف ديال وقت باش نديها في صحتي مزيان,” often emphasizing that health is irreplaceable. These responses reflected higher levels of the construct, characterized by proactive engagement and ownership of health-related decisions. In contrast, a more reactive than anticipatory orientation toward health was also evident. Some participants described competing socioeconomic demands that constrained their ability to prioritize health, as illustrated by “خصك تجيب القوت اليومي لولادك، يعني مكتفكرش في الصحة ديالك” (You must feed your children every day, which leaves little room to think about your own health). These responses highlight a clear differentiation between the upper and lower levels of the concept, indicating that the item’s intention, recognition of personal responsibility, and active involvement in one’s own health were well understood, while revealing contextual factors that may limit their implementation.

#### Social support for health

The items within this scale were identified as the most comprehensible by participants. A majority of respondents reported having strong emotional and practical support networks, particularly within their families, with women playing a crucial role in health-related assistance. The phrase “الزوجة ديالك الأم ديالك و الحمد لله” (“Wife and mother, thanks to God”) exemplifies this. Only minor lexical adjustments were necessary to convey warmth and familiarity consistent with local cultural norms. Some participants questioned whether this support was material or moral. Many expressed, “العول على سيدي ربي; الحمد لله الناس واقفين معانا فجنبنا ماديا ومعنويا” (“Ask help from God. There are always people who help materially and morally”).

#### Appraisal of health information

While generally comprehensible, the items on this scale indicated that participants perceived a limited ability to assess the reliability and accuracy of health information. Many participants reported using the internet to seek health information but struggled to apply clear criteria to evaluate the credibility of sources, with trust in healthcare professionals remaining predominant. To enhance conceptual clarity, the phrase “health information from different sources” (المعلومات الصحية من مصادر مختلفة) was simplified to “المعلومات على الصحة من جوايه مختلفة” (“health information from various places”). Regarding items assessing the evaluation of multiple, reliable sources of health information (Scale 5), participants frequently reported consulting various sources (“كنشوف بزاف ديال البلايص”), including Google, official websites, and reputable doctors (“اللي قريتي عليها ولا جبدتيها من مواقع رسمية ودكاترة كتعرفهم”). Participants further distinguished between information obtained from the internet and that from “الناس دالصحة” (health professionals). These observations are consistent with the constructs intended by the scale, capturing both the diversity and perceived credibility of health information sources, while also identifying a potential area for enhancing critical health literacy.

#### Ability to actively engage with healthcare providers

Cultural norms pertaining to medical authority have influenced participants’ responses to this scale. Many conveyed that they did not engage in “talking with” their physicians, instead simply responding to questions. As a result, the verb “to discuss” (“تْناقش”) was substituted with a more neutral term meaning “to ask” (“تْسوّلْ”). Engagement remains heavily dependent on the relational dynamics and the healthcare provider’s availability.

Qualitative feedback on this Scale, predominantly from female participants, unveiled themes of restraint and silence in healthcare interactions: “You cannot complain about them; you keep that between you and yourself”, *“Some individuals are shy and may suffer in silence without seeking treatment”*, and *“It is challenging to express oneself, perhaps due to reserve or shyness, and a reluctance to discuss personal issues”* (Table 3). Although these *accounts* cannot be conclusively linked to gender, the scale’s emphasis on female participants and themes of modesty suggests gender-specific barriers to health communication.

#### Navigating the healthcare system

The items on this scale were generally the most difficult to understand. Participants described multiple barriers (structural, geographic, financial, organizational) and struggled with abstract terms like “health service” (adjusted from “الخدمات الصحية” to “الخدمات ديال الصحة”). The majority of respondents requested clarifications; for instance: “غالبا كنمشي للمستوصف دالحومة هما كايقولو لي فين نمشي” (which translates to: “Most of the time, I go to the neighborhood primary care center, and they direct me where to go”), exemplifying navigation confined to readily accessible local resources. Despite these challenges, participants articulated clear views on healthcare access, often defining it as the choice between public and private facilities and focusing on medication availability and physician presence. Regarding the item on perceived rights to health services (Scale 7, item 39), respondents engaged directly with the concept of rights. For instance, some stated, “كنظن كاع اي حاجة كتخص الصحة ديالي عندي فيها الحق” (“I believe that anything related to my health, I have the right to it”), while others explicitly denied possessing such rights. Cross-referencing these responses with the participants’ sociodemographic profiles indicates that this divergence is not incidental. Participants who affirmed their rights were predominantly urban residents, formally employed, covered by institutional health insurance, and possessed university-level education. Conversely, those who denied their rights were more likely to be uninsured, reside in rural areas, have low or no formal education, and be outside the formal labor market.

#### Ability to find good health information

The items on this scale initially posed comprehension challenges for participants, primarily because distinguishing credible sources remained difficult despite the widespread use of social media and the internet. These issues were systematically addressed during the translation process, notably by adapting the phrase “health information” from “المعلومات الصحية” to more natural Darija equivalents, such as “معلومات على الصحة,” thereby improving accessibility across comparable scales. Participants consistently identified health professionals, namely pharmacists, doctors, and nurses, as the most trusted sources, while also acknowledging online forums and testimonials. One participant remarked: “بالنسبة للناس الكبار واللي ما كيعرفوش يقرأوا ويكتبوا، راه صعيب عليهم” (“For elderly individuals and those who are illiterate, it is difficult”), underscoring the importance of human support and platforms that utilize the Darija language.

#### Understand health information well enough to know what to do

The initial translations for this scale posed challenges, particularly for participants with limited literacy. Even literate individuals showed little confidence in reading medical documents or understanding instructions, especially those written in standard Arabic, which differs significantly from everyday language. Many relied on healthcare professionals or administrative staff for clarification. Additionally, participants consistently underscored the vital role of community pharmacists in clarifying prescriptions, medication use, and written materials. Three respondents explicitly stated, “*It is the pharmacist who explains things clearly to me.*”.

All participants emphasized that they strictly followed healthcare professionals’ instructions; one said, “كنسمع ليهم وكنفّذ بلا زيادة بلا نقصان” (“I do exactly what they tell me, without adding or subtracting anything”).

## Discussion

This research aimed to translate and culturally adapt the widely used HLQ and to verify whether diverse respondents understood and answered items as intended. Using the Translation Integrity Protocol (TIP), our expert panel, including Moroccan primary health care users, encountered challenges translating English concepts into Darija, given its spoken nature and multilingual influences (Classical Arabic, Amazigh, French, and Spanish). In-depth discussions confirmed the cultural relevance of the concepts. Based on the detailed intentions of the HLQ items and scale descriptors, the team examined Darija nuances, incorporating input from community members and health professionals to develop the interpretability of scores in the Moroccan context [13, 15–19, 22, 41, 44–47].

Of the 28 diverse participants (median age 39; predominantly female), the sample included two distinct groups: young adults with higher education and older adults with less education, accurately reflecting the profile of primary care consultants in Morocco. The most revised scales pertained to engagement with health professionals, system navigation, and understanding health information. Participants generally demonstrated good comprehension, though qualitative analysis uncovered critical nuances: trust in medical authorities was closely linked to passive dependence, and “health management” was frequently interpreted as mere compliance rather than a proactive approach. Participants also reported difficulties navigating the system and accessing understandable health information. Social support emerged as the most easily understood scale, reflecting the central role of family networks in the Moroccan health context.

### Validity and cultural adaptation of HLQ items

The cognitive interview process indicated that the conceptual framework underlying the nine HLQ scales is broadly applicable to the Moroccan context. Of the 44 items, 25 were confirmed as conceptually equivalent without modification, while 19 required linguistic or cultural refinement following the consensus meeting. The most frequently revised scales concerned engagement with healthcare professionals, access to the healthcare system, and understanding of health information. This pattern reflects real structural barriers rather than simple translation difficulties and aligns with findings from other studies on adapting the HLQ across various linguistic and cultural contexts. The current results confirm and expand on data from HLQ adaptations in Francophone and culturally similar contexts. The French adaptation carried out in metropolitan France [48], focused on diabetic populations at cardiovascular risk attending community health education programs. Particular attention was given to concepts related to engagement in care and access to the healthcare system, whereas items related to social support were among the most readily understood. Another study, using cognitive interviews, tested the validity of the French HLQ with migrant populations, many of whom were of North African origin [16]. The study revealed that the vast majority of items were comprehended as anticipated, even by participants with limited educational backgrounds and varying levels of proficiency in French. Nevertheless, certain words or concepts presented challenges for the least educated participants. The heterogeneous health literacy profiles observed within this migrant population reflect the diversity observed among Moroccan users of primary health care, where education level, language skills, and familiarity with the formal health system differ significantly [49]. In the Francophone sub-Saharan context, this study offers another valuable comparison [50]. It emphasizes the importance of grounding item adaptation in the concrete experiences of populations within health systems characterized by the legacy of colonial language, limited resources, and a strong reliance on community and family support, challenges that are comparable to those faced during adaptation in Morocco. Outside the Francophone world, the Arabic adaptation in Jordan [18] aligns with the TIP and is the most relevant linguistic comparison. It demonstrates that the HLQ’s nine conceptual domains can be effectively translated for an Arabic-speaking audience. However, the Darija context presents additional complexity, as Moroccan Darija is a spoken dialect distinct from Modern Standard Arabic and written Arabic, requiring adaptation decisions that go beyond translation to address fundamental issues of register, orality, and cultural resonance.

In the German, Portuguese, and Vietnamese adaptations [17, 40, 51], the scales measuring engagement in care, system orientation, and information comprehension required careful translation and cultural adaptation. They reflect patients’ real experiences with structural and communication barriers, rather than mere linguistic artifacts. Despite linguistic and healthcare system differences, the difficulties encountered in adapting these HLQ scales to Darija (Moroccan Arabic) point to genuine health literacy issues rather than errors in the adaptation process. Qualitative analysis revealed that participants’ responses aligned with the descriptions and concepts of the HLQ scales, even when initial comprehension was difficult. These findings confirm the conceptual relevance of the HLQ items in Darija and contribute content and response processes validity evidence to a validity argument about the use of the Darija HLQ in Morocco [9, 52, 53].

### Sociocultural representations of health

The cognitive interviews provided valuable qualitative insights into how Moroccan primary care users understand and experience health literacy in their daily lives. Three interdependent themes emerged: the relationship with medical authority, the central role of social and family support, and the gendered dimensions of access to care.

The most prominent discovery in this context was the recurrent confusion between health management and passive obedience to healthcare professionals’ instructions. Participants consistently perceived managing their health as merely following directives rather than as an active, autonomous process. This inclination exemplifies a form of interpretive dependence that is closely associated with trust in medical authority. Such functional health literacy, defined by the capacity to receive and utilize information, does not encompass the critical analysis skills inherent in advanced levels of health literacy. These results argue for targeted interventions that, beyond simply transmitting information, aim to develop interactive and critical health literacy skills [54] while simultaneously strengthening the adaptability of healthcare structures to the linguistic and cultural realities of their populations. Scale 4. Social Support for health was remarkably the most comprehensible for all participants, thus emphasizing the pivotal role of family and social networks as facilitators of health education in Morocco. This observation corroborates the collective and family-oriented approach to health decision-making as documented in Maghreb and Middle Eastern contexts, where family members habitually accompany patients, exchange information, and serve as advocates during medical consultations. Going forward, the use of the Darija version of the HLQ should capitalize on this strength by using data from Scale 4 to identify populations lacking adequate social support for health education, thereby enabling the development of targeted community-based interventions.

An important yet less frequently discussed aspect of healthcare access involves pharmacists. Cognitive interviews indicated that pharmacists are vital facilitators at the intersection of patients and the healthcare system, serving as interpreters and intermediaries, especially because medical prescriptions are primarily written in French. This language barrier stems from Morocco’s historical healthcare training, traditionally conducted solely in French, and from the common practice of self-medication, in which patients often consult pharmacists directly without medical advice. Consequently, pharmacists play a crucial role in the patient care network. This must be considered when interpreting the HLQ scales related to care access (scale 7) and interaction with healthcare providers (scale 6), especially when patients cannot read the Modern Standard Arabic leaflets on medication packages. Future studies and programs in Morocco focused on health education should explicitly recognize the pharmacist’s role as both a resource in this area and a structural facilitator.

Gendered dimensions were also emphasized in the cognitive interviews on health literacy, which necessitate particular attention. Participants, notably, reported difficulty in discussing their health issues with healthcare professionals, stating, for example: “Some people are shy and may be suffering without daring to ask for treatment” and “It’s difficult to speak up, perhaps because you’re reserved or shy and don’t want to talk about your problems.” These statements, directly extracted from the cognitive interviews conducted as part of this study, highlight the influence of modesty, gender-related power dynamics, and social norms in restricting women’s access to healthcare and their capacity to articulate their needs [55]. This phenomenon has been observed in other contexts, notably in Mali and among North African migrant populations [56–58]. In a country where illiteracy affected 42% of adult women and 77% of literate men in 2024, and where women are overrepresented among those with low levels of education, these obstacles are compounded by structural inequalities. Although the methodology of this study does not permit a systematic comparison of responses between men and women in the cognitive interviews, the qualitative data robustly support subsequent analysis that stratifies HLQ scores by gender in order to identify the areas where health literacy interventions are most effective in mitigating these inequities [25, 59].

### Implications for practice and research

The availability of the Darija HLQ now facilitates population-level health literacy profiling, which can directly inform needs assessments and the development of interventions using the Optimising Health Literacy and Access (Ophelia) process [60, 61].

For healthcare professionals, the tool’s nine domains facilitate a strengths-based diagnostic approach that recognizes both individuals’ challenges and their strengths. The observation that social support (scale 4) is a reliable and comprehensible dimension within this population indicates its potential to serve as a leverage point for health literacy interventions aimed at mobilizing family and community networks. Conversely, the ongoing challenges observed in system navigation (scale 7) and in engagement with healthcare professionals (scale 6) highlight the need for organizational strategies to enhance communication and support for Darija-speaking patients within healthcare services. This need is especially pertinent given the crucial role pharmacists often play as de facto intermediaries in health literacy. For researchers, these findings support using the Darija version of the HLQ in quantitative surveys, clinical trials, intervention evaluations, and needs assessments across chronic disease and primary healthcare settings. Future research should examine the relationship between subpopulation clusters and specific health outcomes, such as medication adherence, preventive service utilization, chronic condition management, and emergency department visits, with particular emphasis on analyses stratified by sex. The linguistic validation data also contribute to the international literature on the TIP [41]. Therefore, enhancing its utility as a methodology for producing instrument versions that are both linguistically accurate and conceptually equivalent across typologically diverse languages.

For policymakers, Morocco’s multilingual context, in which Darija is spoken by the majority of the population, yet official health communication is predominantly in French and Modern Standard Arabic, poses a structural challenge to health literacy that cannot be addressed solely at the individual level. The Darija HLQ provides evidence supporting advocacy for linguistically sensitive healthcare, including developing patient education materials in Darija, training healthcare professionals to communicate with Darija-speaking patients, and systematically integrating community health workers and pharmacists as health literacy resources within primary care settings. These recommendations align with calls for simultaneous intervention to improve individual health literacy skills and the organizational adaptability of healthcare facilities [62].

## Strengths

A significant advantage of this research is its rigorous methodology for translating and culturally adapting the HLQ into Moroccan Darija. This process included multidisciplinary consensus meetings with researchers, translators, and healthcare professionals, enhancing the adapted tool’s cultural and conceptual relevance.

Conducting 28 face-to-face cognitive interviews enabled a thorough investigation of how participants understood and responded to the items, capturing their spontaneous interpretations, emotional responses, and possible misconceptions. This method helped identify necessary modifications to ensure the instrument was conceptually equivalent in the Moroccan context. Moreover, the depth of the qualitative data collected provided insights that quantitative methods often miss, particularly regarding how health-related concepts, interactions, and terminology are perceived and articulated in a predominantly oral language like Darija.

Lastly, the use of an inductive thematic analysis, rooted in the HLQ conceptual framework, provided a thoughtful examination that remained true to the original instrument while remaining attuned to the contextual differences in health literacy experiences.

## Limitations

This study has some sample-related limitations. The primary limitation was that participants were recruited from only two provinces; the findings might not fully reflect Morocco’s geographical and linguistic diversity, especially among Amazigh-speaking communities. Additionally, women made up a larger share of the sample. While this mirrors national trends in healthcare use and education, it could have shaped the perspectives included. Certain groups, such as people in remote rural areas or those with limited contact with healthcare, may not have been adequately represented.

Another limitation of the context interviews is that they were conducted in healthcare settings, which may have affected how openly people shared their thoughts, especially when discussing their experiences with health professionals. Some participants might have experienced pressure to provide positive responses or to withhold criticisms, which could result in incomplete expression of all concerns or misunderstandings. Finally, this qualitative phase concentrated on evaluating the linguistic, cultural, and conceptual equivalence of the HLQ items. Further research is needed to evaluate the psychometric properties of the Darija HLQ in a larger, more diverse sample. Such validation will be necessary to examine the instrument’s internal structure and reliability in the Moroccan context.

## Conclusion

The adaptation of the HLQ into Moroccan Darija confirmed the relevance of the nine scales and highlighted key aspects of the local population’s health relationship. The process emphasized a participatory, contextualized approach to maintain the instrument’s integrity and cultural fit. The adaptations improved the Darija HLQ content within Morocco, making it more sensitive to linguistic, social, and relational realities. It is now ready for psychometric testing in a larger group of primary care users to evaluate its use as an evidence-based tool to guide public health, research, and health education in Morocco. The strong foundations, fostered by its participatory cultural adaptation, show potential to incorporate community perspectives and promote justice in health promotion and policy. This validity study provides strong evidence of its cultural and conceptual suitability in Morocco. Future research will evaluate its psychometric properties in a representative Moroccan sample, advancing health literacy measurement and tailored interventions to reduce health inequities.

## Electronic supplementary material

Below is the link to the electronic supplementary material.

Supplementary material 1

## Data Availability

The datasets generated and/or analyzed during the current study are available from the corresponding author on reasonable request.

## Declarations

### Declaration of AI-assisted writing tools (corresponding author)

The corresponding author reports utilizing ChatGPT to assist with translating selected sections of the manuscript and employing Grammarly to enhance clarity, grammar, and readability. These tools were used solely for linguistic and editorial support. All scientific content, interpretations, and conclusions were conceived and validated by the authors. The corresponding author assumes full responsibility for the integrity and accuracy of the manuscript.
