# Supplementary material for: Translation and cultural adaptation of the health literacy questionnaire (HLQ) in Morocco
Source: J Patient Rep Outcomes. 2026 Jun 11;10:149. doi: 10.1186/s41687-026-01111-3 (PMC13486425; doi:10.1186/s41687-026-01111-3)
Supplement: Supplementary file 1 [file 41687_2026_1111_MOESM1_ESM.docx]

**Step 1: Forward Translation (English****Darija)**

***TIP Translation Management Grid***

| Translator YI Native Darija speaker, fluent in English Forward translation with item intent descriptions | Translator AL Native Darija speaker, fluent in English Forward translation with item intent descriptions | Consensus most suitable translation  was agreed |
| --- | --- | --- |

# ▼

**Step 2 : Backward Translation (Darija** → **English)**

Translator SN Native English speaker, health sciences background

*Blind to the original English HLQ and item intent descriptions*

▼

**Step 3: HLQ Developer Review**

RO (HLQ co-developer) reviews back-translation via the Translation Integrity Protocol (TIP) **Management grid**

Feedback and comments provided to translators

▼

**Step 4: Translation Consensus Meetings (7 hours, 2 sessions)**

12 participants: 2 researchers (KB, MC) · 3 translators · 4 community members · 1 educator · 2 healthcare professionals Guided by HLQ item intent descriptions

*Conceptual fidelity + Cultural-linguistic appropriateness*

**Recommended final Darija HLQ version**

▼

| **Step 5: Cognitive Interviews (Field Testing)**  In-person: Observation of non-verbal cues and emotional responses Think-aloud protocol: Reading Interpreting Verbalizing  Answering the structured notes template + Audio recording | | |
| --- | --- | --- |
|  | | |
| Transcription & verification | Translation: Darija French / English | Cross-linguistic analysis |

**Figure 1. HLQ translation and cultural adaptation process into Moroccan Darija, informed by the TIP Management Grid framework (Osborne & Hawkins).**
